# Comparison of Nutrigenomics Technology Interface Tools for Consumers and Health Professionals: Protocol for a Mixed-Methods Study

**DOI:** 10.2196/resprot.9846

**Published:** 2018-06-11

**Authors:** Paula Littlejohn, Irene Cop, Erin Brown, Rimi Afroze, Karen M Davison

**Affiliations:** ^1^ Department of Microbiology and Immunology University of British Columbia Vancouver, BC Canada; ^2^ Health Science Department of Biology Kwantlen Polytechnic University Surrey, BC Canada; ^3^ Fraser Health Authority Surrey, BC Canada; ^4^ Vancouver Coastal Health Vancouver, BC Canada; ^5^ Department of Psychology University of Hawai`i at Mānoa Honolulu, HI United States

**Keywords:** nutrigenomics, nutrigenetics, genomics, epigenomics

## Abstract

**Background:**

Although nutrition interventions are a widely accepted resource for the prevention of long-term health conditions, current approaches have not adequately reduced chronic disease morbidity. Nutrigenomics has great potential; however, it is complicated to implement. There is a need for products based on nutrition-related gene test results that are easily understood, accessible, and used.

**Objective:**

The primary objective of this study was to compare a nonpractitioner-assisted direct-to-consumer self-driven approach to nutrigenomics versus an integrated and personalized practitioner-led method.

**Methods:**

This 4-month study used a mixed-methods design that included (1) a phase 1 randomized controlled trial that examined the effectiveness of a multifaceted, nutrition-based gene test (components assessed included major nutrients, food tolerances, food taste and preferences, and micronutrients) in changing health behaviors, followed by (2) a qualitative investigation that explored participants’ experiences. The study recruited 55 healthy males and females (aged 35-55 years) randomized as a 2:1 ratio where 36 received the intervention (gene test results plus integrated and personalized nutrition report) and 19 were assigned to the control group (gene test results report emailed). The primary outcomes of interest measures included changes in diet (nutrients, healthy eating index), changes in measures on General Self-efficacy and Health-Related Quality of Life scales, and anthropometrics (body mass index, waist-to-hip ratio) measured at baseline, post intervention (3 and 6 weeks), and the final visit (week 9 post intervention).

**Results:**

Of the 478 individuals who expressed interest, 180 were invited (37.7%, 180/478) and completed the eligibility screening questionnaire; 73 of the 180 invited individuals (40.5%) were deemed eligible. Of the 73 individuals who were deemed to be eligible, 58 completed the baseline health questionnaire and food records (79%). Of these 58 individuals, 3 were excluded either because they did not complete all required data collection forms or were later found to be ineligible. The final sample (n=55) was mostly female (75%), married (85%), and those who had completed postsecondary education (62%).

**Conclusions:**

This study will leverage quantitative and qualitative findings, which will guide the development of nutrigenomics-based products in electronic formats that are user-friendly for consumers and health professionals. Although the quantitative data have not been analyzed yet, the overwhelming interest in the study and the extremely high retention rate show that there is a great degree of interest in this field. Given this interest and the fact that nutrigenomics is an evolving science, a need for continued research exists to further the understanding of the role of genetic variation and its role and applications in nutrition practice.

**Trial Registration:**

Clinicaltrials.gov NCT03310814; http://clinicaltrials.gov/ct2/show/NCT03310814 (Archived by WebCite at http://www.webcitation.org/6yGnU5deB)

**Registered Report Identifier:**

RR1-10.2196/9846

## Introduction

### Background

Globally, chronic disease is a leading cause of death and morbidity that creates an ever-increasing economic burden on health care [1-3]. Diet is recognized as a significant modifiable risk factor in the development of chronic diseases such as diabetes, cardiovascular disease, certain cancers, and depression [4]. However, current nutrition approaches have not been adequate to effect the changes needed. Historically, nutrition science has presupposed that everyone absorbs and metabolizes nutrients similarly, and differences in nutrient requirements vary only by factors such as gender, age, and pregnancy or breastfeeding status [5]. However, one’s nutritional status and the development of complex diseases also depend on the interaction of nutrients with DNA. Nutritional genomics, which encompasses nutrigenomics and nutrigenetics, improves on current health practices by enabling more tailored nutritional advice targeted to individual needs.

Nutrigenetics investigates the effect of genetic variation on nutrient bioavailability and metabolism. Nutrigenomics further investigates how nutrients and bioactive food compounds affect human health through epigenetic modifications [6-10]. For example, exposure to dietary deficiencies or excesses can result in changes in the epigenome, which alters gene expression profiles and other genome functions, leading to physical and mental health deterioration [11-13]. Therefore, by customizing an individual’s dietary intake based on integration of life stage, current health status, and genome information, there is the potential to prevent or ameliorate the effects of conditions such as diabetes, metabolic syndrome, cardiovascular disease, cancer, and depression [7,14-17]. The application of tailoring one’s diet from different information sources that include dietary-related DNA-based results, referred to as personalized nutrition, is becoming increasingly recognized as part of the next paradigm in health practice.

Although the advancement of nutrigenomics and personalized nutrition shows significant promise in improving population health, it also presents challenges. Nutrigenomics is more complicated to understand and deliver than current nutrition intervention approaches. Among health practitioners and government entities, there is concern about direct-to-consumer gene testing, particularly those which examine risk for disease, as it is unclear how individuals perceive and translate the information given that there is no or little involvement from health professionals [18-20]. Busy health professionals, who may want to integrate nutrigenomics as part of their practice, may not have the time to learn the intricacies of the technology and the scientific background or think they have the competencies to explain findings and suggest modifications to individuals. Consumers have been largely left to find the information and interpret the findings themselves, leaving room for misinterpretation and misuse [20]. Despite these current issues, studies have shown that personalized nutrition based on gene test results improve dietary quality [15,21,22]. Therefore, the potential to use nutrition-related genetic information to optimize dietary interventions that can improve lives and health care costs is too great to ignore.

It is thought that to advance the application of nutrigenomics to personalized nutrition is going to require the training of health professionals who can work with consumers to appropriately provide guidance on the gene test results. In addition, there is a need to create technology-based interface tools that integrate currently accepted nutrition guidelines (eg, *Dietary Reference Intakes* [5]), phenotypic information about the person’s current nutritional status (eg, anthropometry, physical activity), and genotype-directed nutrition based on rare or common gene variation [23]. This study proposes to compare standard and tailored personalized nutrition approaches based on gene testing and to elicit participant feedback about their experiences with the 2 types of interventions. The study results will be leveraged to generate new and tailored nutrigenomics tools that are digitally based for consumers and health professionals.

### Objectives

The overall goal of this study was to investigate whether personalized dietary advice based on genotypic testing provided by a practitioner leads to greater dietary improvements and health outcomes compared with a nonpractitioner-assisted direct-to-consumer (DTC) self-driven approach. The main study objective was to compare a practitioner-facilitated personalized dietary approach that uses genotypic and phenotypic information with a DTC self-driven approach and their impact on changing participant’s knowledge, motivation, and behavior related to diet and eating and the quality of their diet. It was hypothesized that significantly higher levels of knowledge, motivation, and behavior would be reported, and there would be increased diet quality changes in the group that receives personal DNA diet information and customized dietary advice (practitioner-led) compared with the group that is provided personal DNA diet information (DTC self-driven approach) only. In addition to this primary objective, changes in self-efficacy were evaluated to determine whether it was a potential mediator/moderator of dietary changes, and changes in quality of life were assessed as a possible additional benefit to dietary changes. Focus group interview data were also collected to explore participants’ experiences with using personalized nutrition tools and resources.

## Methods

### Study Design

A mixed-methods study was conducted, consisting of 2 stages: (1) an exploratory randomized controlled pilot study (2:1 allocation ratio) comparing standard DTC self-driven versus practitioner-facilitated approaches that use DNA-based diet information and (2) qualitative investigation of participants’ experiences to examine the feasibility and acceptability of the intervention. The study protocol, including paper-based or Web-based data collection forms, was approved by Quorum Institutional Review Board (protocol #32220CDN/1). All participants were required to provide informed consent before enrolling in the study (online). The initial online consent form outlined the details of the study and requested consent to collect eligibility screening information and if eligible consenting to provide baseline information. A time estimate of 15 to 20 min was indicated for completing the baseline questionnaire that was based on pilot testing of the online survey with study investigators and student volunteers (n=11). A second written consent form was reviewed at the first site visit with the participant, and they were invited a second time to consent to continued involvement in the study. On this questionnaire, they were given time estimates of 30 to 60 min for each onsite visit and 15 to 20 min to complete the online questionnaires between visits. The protocol was registered with the U.S. National Library of Medicine (trial registration #NCT03310814).

### Study Participants and Setting

Participants included adults (aged 35-55 years) who were deemed eligible based on various criteria (Textbox 1). This age range was selected as people tend to typically notice changes in their health [24]. Sample size determination was based on estimated mean differences in diet quality scores used in a personalized nutrition intervention study [21], application of sample size for 2-sample comparison of means for repeated measures [25], and estimated rate of loss to follow-up of 10% (3/32). Using similar approaches and previous study results [26,27], we determined we would need a minimum of 16 participants per group to detect differences in self-efficacy and quality of life.

**Study participation: inclusion and exclusion criteria.**

Inclusion criteria

1. Adults, aged 35-55 years
2. Ability to understand, sign an informed consent, and to provide a buccal DNA swab
3. Willing to improve health
4. Medically stable. Subjects with diet-related chronic disease can enroll provided that their condition was stabilized or well controlled for at least 6months at the time of the baseline visit

Exclusion criteria

1. Currently on a therapeutic or restrictive diet (eg, Atkins)
2. Diagnosis of 2 or more chronic diseases or unstable chronic disease as deemed by accepted clinical guidelines
3. Clinical diagnosis of any mental health condition
4. Any of the following conditions: HIV; chronic obstructive pulmonary disease; severe/uncontrolled asthma; cystic fibrosis; bronchiectasis; interstitial lung disease; chronic renal failure; colon or small intestine problem; liver or kidney disease; uncorrected hypothyroidism or hyperthyroidism in the previous 12 months; alcohol or drug dependence during the previous 12 months; current or former malignancy for which the participant has undergone resection, radiation therapy, or chemotherapy within previous 5 years
5. Currently enrolled or plan to be enrolled in another research study during the course of the investigation
6. Planned or recent (within the last 12 months) bariatric surgery
7. Current use of weight-altering medication for the purpose of weight loss
8. Investigators and their immediate families, with immediate family defined as a spouse, parent, child, or sibling, whether biological or legally adopted
9. Pregnant and/or breastfeeding
10. Current smoker
11. Body mass index ≥35
12. Any other health risk or condition that may put the participant at risk, or influence the results of the study or the participant’s ability to participate in the study

### Description of Study Groups

Participants, who were deemed eligible and provided consent, were randomized by a statistician independent to the study into either the intervention or control group. Those randomized to the intervention (I) group received their gene test result report (standard) and an integrated report in paper and online format that integrated information about their gene tests, dietary intakes in relation to the standards, and personalized DNA-based diet plan as recommended by current guidelines regarding personalized nutrition [23]. They also received counseling by a trained research registered dietitian (RD).

The counseling provided by the RD was based on both reports outlining their genes, markers, and variants. It included a corresponding DNA-based diet recommendation. For example, those who possessed the genotype that has been associated with increased risk of a health outcome were provided a “targeted” dietary recommendation. Subjects not possessing the specified risk variant received the current standard dietary recommendations [5,28]. In addition, the RD worked collaboratively with the participant to define 1 to 3 nutrition-related goals they would work on. Both groups received 3 follow-up emails (one every 2 weeks post intervention) with information about nutrigenomics as well as tips and reminders (eg, information about label reading) to help them reach their nutritional goals.

After the study is complete, participants randomized to the control (C) group will receive the intervention, that is, they will receive DNA-based dietary advice at the final study visit. Therefore, they will receive the same benefits, if any, as those who had the full intervention.

### Study Visits

**Figure 1:**
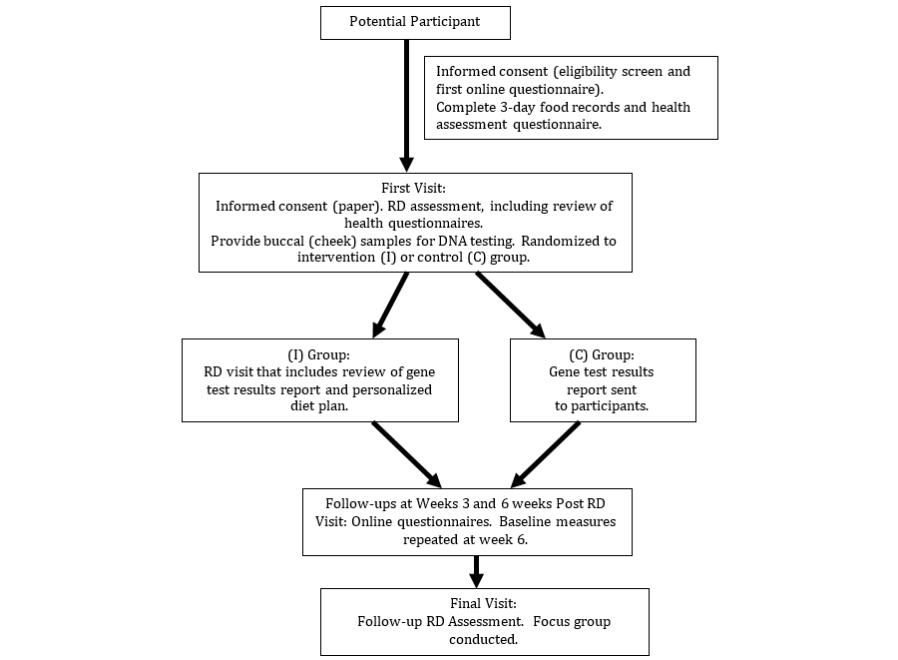
Overview of study design. RD: registered dietitian; I: intervention group; C: control group.

The study was conducted over 4 months and consisted of 6 points in time where participants either visited with research team members or provided information via online questionnaires (Figure 1). The online closed questionnaires (only study participants could access) were developed in FluidWare’s FluidSurveys [29] and in accordance with the Checklist for Reporting Results of Internet E-Surveys [30]. The online questionnaires were developed using standard measurement tools (see section Measurements) and protocols for nutrition assessment [31,32]. All data collection tools were pilot-tested among the study staff and student volunteers (n=11) for usability and technical functionality. For each online questionnaire, the numbers of pages (screens) were 12 or less, and participants could navigate it using back buttons and review functions. Participants were emailed instructions and the links to each online questionnaire at the appropriate time in the delivery of the study protocol. The study coordinator checked each questionnaire for completeness and for duplicate entries and followed up with participants as needed. In instances where participants had more than one questionnaire filled out, the most recent entries were used for analysis.

All online data were collected and stored in accordance with QUORUM guidelines to protect unauthorized access. A schedule of events is provided in Table 1.

#### Recruiting/Screening: Online

Participants who entered the screening phase electronically signed an online informed consent form to grant permission to collect eligibility information and baseline health information (if deemed eligible). If the participant met the inclusion criteria, they were sent a 3-day food record to complete within 7 days (±3 days) of their first site visit. In addition, they were sent the link to complete an online baseline assessment questionnaire that collected information about sociodemographics, their health (eg, presence of any health conditions, medications, supplements used), health-related quality of life (Short-Form 8, SF8), self-efficacy (General Self-Efficacy, GSE), physical and sedentary activities, food intakes (food frequency, food selection), and sleep quality.

#### Baseline Physical (On-Site) Visit

At the first visit, each participant met with the research team. They reviewed and signed a paper copy of the informed consent. The RD studied their online questionnaire information and did a nutrition assessment. Baseline measures of height, weight, and waist and hip circumference, based on standardized protocols [32], were performed. The 3-day food records were also reviewed to ensure completeness and accuracy.

At this stage of the study, a buccal swab cheek sample was obtained for gene testing. Buccal DNA samples were collected using Oracollect-DNA OCR-100 swabs (DNA Genotek, Ottawa, Canada). The RD collected the samples, identified with barcodes for confidentiality and blinding, and stored them between 15°C and 30°C. Participants did not eat or drink at least 30 min before obtaining their buccal swab. The samples were processed at the Clinical Genomics Centre at Mount Sinai Hospital in Toronto using Agena MassARRAY. Gene testing was done in 5 areas (Table 2) that were selected based on recommended evidence approaches to personalized nutrition [33].

Participants were informed that the processing time to receive results would be approximately 2 to 4 weeks. Between visits 2 and 3, a statistician independent to the study completed the randomization to the I and C group, using a computerized random generator.

### Test Results and Diet Plan

Approximately 4 weeks (±3 days) following the baseline visit, participants in the I group received a consultation visit with the RD who reviewed their individualized diet plan tailored to their health information and gene test results. Those in the C group were emailed their DNA test results report. Collection of information about adverse events, change in health status since the baseline visit, and concomitant medication was obtained.

**Table 1:** Schedule of events.

Activity	Screen	V1^a^	Baseline (V2)	Consult (V3)	Follow-up assessments			Follow-up^b^
					4 weeks post consult (V4)	8 weeks post consult (V5)	Final (V6)	
Informed consent	✓^c^	—	✓^d^	—	—	—	—	—
Eligibility screen	✓^c^	—	—	—	—	—	—	—
Food records	—	✓	—	—	—	✓^c^	—	✓
Baseline^e^	—	✓^c^	✓^f^	—	—	✓^c^	✓^f^	✓^f^
Registered Dietitian assessment	—	—	✓	—	—	—	✓	—
Anthropometrics	—	—	✓	—	—	—	✓	—
DNA buccal cheek swab	—	—	✓	—	—	—	—	—
Consults: report^g^ & Report + DNA-based diet advice^h^	—	—	—	✓	—	—	✓^g^	—
Follow-ups^i^	—	—	✓	—	✓^c^	✓^c^	—	✓
Focus groups	—	—	—	—	—	—	✓	—

^a^V: visit.

^b^4 weeks post final visit; control group.

^c^Online form.

^d^Paper form.

^e^Baseline: sociodemographics, Short-Form 8 (health-related quality of life), General Self-Efficacy, physical and sedentary activities, sleep quality (HealthMeasures’ Patient-Reported Outcomes Measurement Information System ), Food Frequency and Selection Questionnaire.

^f^Information reviewed.

^g^Control group receives intervention.

^h^Intervention group.

^i^Follow-ups: income, social support, knowledge, behavior, action, adverse events, and concomitant medications.

**Table 2:** Description of gene test.

Area measured	Nutrient or food component tested
Diet management	Carbohydrates; cholesterol (high-density lipoprotein); cholesterol (low-density lipoprotein); fat—dietary; fat—stored; fat—monounsaturated; fat—saturated; insulin; protein
Weight response	Body mass index
Food tolerances	Alcohol; caffeine; gluten; lactose; salt; sugar craving
Food taste and preferences	Caffeine; carbohydrate; fat preference; protein preference; bitter taste; salt taste; sweet taste
Vitamins, minerals and essential fats	Vitamin A; vitamin B_6_; vitamin B_9_ (folate); vitamin B_12_; vitamin C; vitamin D; vitamin E; calcium; iodine; iron; omega 3; omega 6

### Three- and Six-Week Online Check-Ins

At 3- and 6- weeks post intervention, both participants in I and C groups were sent their first follow-up online questionnaire with baseline measures repeated. Information about any adverse events (AEs), change in health status, and concomitant medication information was obtained. The only difference between the 2 questionnaires was that the I group questionnaire asked about whether knowing one’s personal DNA helped the participant choose specific foods and meals to eat healthier. After the week 6 check-in, participants were sent food records to complete and bring to the final on-site visit. At the time of writing, the study is just in the completion of this phase.

### Final Visit

At the final visit, the research team will review all data collected post intervention to ensure accuracy and completeness. The research dietitian will then conduct a repeat nutrition assessment. Participants in the C group will receive an individualized diet plan tailored to their health information and gene test results. Invitations to attend a focus group to solicit feedback about their experiences with the study will be extended. The interview guide for the focus group concentrates on collecting data about participants’ responses to their gene test results and RD consultation, how they used their results, suggestions for improvements, and impressions about barriers and facilitators in using their results.

### Outcome Measurements

All online measurement tools were pilot-tested with upper level university students and faculty in a science program. The outcome measures are described as follows:

#### Nutrition Outcomes

Three-day food records measured pre- and postnutrient intakes including daily eating patterns. Changes in caloric, macronutrient, micronutrient, and food groups will be measured and compared with national standards (eg, *Eating Well with Canada’s Food Guide*, *Dietary Reference Intakes*). Changes in overall diet quality will be assessed using the Canadian version of the Healthy Eating Index [34]. Protocols for food record data collection will be derived from Health Canada nutrition survey procedures [35].

In addition to nutrient intake information collected from the food records, a food frequency questionnaire (FFQ) is included to assess for usual intakes and to validate the food record information. The food frequency measurement tools were derived from Health Canada nutrition survey measures [35].

Nutrition measures also included food selection questions about types of food selected, dietary restraint, food insecurity, motivation to change diet, and eating behavior changes. These were based on validated measures such as the Three Factor Eating Questionnaire [36], Health Canada, Statistics Canada (eg, the Canadian Community Health Survey), and BC Ministry of Health surveys [35], as well as review of the research literature about measurements of motivation and dietary change and eating behavior changes. Some of these questions were developed by the research team and pilot-tested for comprehension and face validity.

#### Quality of Life and Self-Efficacy Outcomes

Measures of quality of life and self-efficacy were included to assess whether receiving DNA-based dietary information impacted one’s general outlook and confidence to initiate changes. The measurement tools included the following:

##### Health-Related Quality of Life (HRQOL) SF-8 (Short Form 8)

The HRQOL-SF8 is a validated health survey that measures quality of life, functional health, and well-being; the 2 major scales, physical health and mental health, are included. The HRQOL-SF8 has well-established psychometric properties [37] and contains 8 items with a 4-week recall period. Each item has a 5- or 6-point response range. Physical component summary (PCS) and mental component summary (MCS) measures are calculated by weighting each SF-8 item using a norm-based scoring method given in the instrument guidelines. Higher summary PCS and MCS scores indicate better health. Scores above and below 50 are considered above and below the average in general populations [38].

##### General Self-Efficacy

The GSE is a 10-item self-report measure of self-efficacy, the belief in one's competence to cope with a broad range of stressful or challenging demands [39]. It includes questions about one’s perceptions in the ease in which they stick to their aims and accomplish goals and to solve most problems if they invest the necessary effort. The GSE was included as a potential mediator/moderator variable related to any diet changes. It is a validated health scale correlated to emotion, optimism, and work satisfaction. Negative coefficients correlated to the GSE include depression, stress, health complaints, burnout, and anxiety. High reliability, stability, and construct validity of the GSE scale have been confirmed, and Cronbach alphas obtained for the GSE scale have ranged from .86 to .94 [40].

##### Measures of Change in Knowledge, Motivation, and Behavior

Three questions developed by the research team were included to assess for changes in knowledge, motivation, and behavior related to DNA-based dietary advice. These were based on review of the research literature and included questions about the stages of change model [41]. The questions were pilot-tested before use.

##### Anthropometrics

Baseline measures of height, weight, waist circumference, and hip circumference based on standardized protocols [35] are included. Body mass index (kg/m^2^) and waist-to-hip ratio will be calculated.

##### Covariates

Other relevant measures that can influence dietary intake and health behavior were assessed and controlled for. These included:

1. *Natural health product (NHP) usage:* NHP use (eg, vitamins, minerals, botanicals), which can influence nutrient intakes, is recorded at all study time points and included type, dose, and frequency of use. Participants were advised at baseline to keep any NHP use at the same dose and frequency throughout the study.
2. *Physical and sedentary activities:* To measure activity level, physical activity index (PAI) [42] was used. The PAI included questions about the frequency, duration, and intensity of participation in certain activities in the previous 3 months. The data on physical activity were combined to obtain the PAI, which represents the average daily energy expended on leisure-time physical activity, expressed in kilocalories per kilogram body weight per day. To calculate this index, the energy expenditure (EE) for each activity was first estimated (see Multimedia Appendix 1 for details). The overall EE totals are used to categorize individuals as inactive (PAI <1.5 kcal/kg/day), moderately active (PAI 1.5 to <3 kcal/kg/day), and active (PAI ≥3 kcal/kg/day).
3. *Sleep quality*: The Patient-Reported Outcomes Measurement Information System Sleep Disturbance scale-short form [43] is used to assess sleep quality. The instrument is an 8-item self-rated questionnaire, which assesses sleep quality over the previous 7 days. Individual items are scored on a scale from 1 to 5, and scores are summed to yield a total raw score between 8 and 40, with lower scores indicating better sleep or a lesser degree of sleep-related impairments.
4. *Stress*: Because one’s ability to deal with stress can impact dietary intake, 2 validated questions from the Canadian Community Health Survey [44] are included as covariates.
5. *Sociodemographics*: Standard determinants of health are measured that include sex/gender, age, relationship status, income, race/ethnicity, and perceived social support. The questions have been previously validated in studies such as the Canadian Community Health Survey.

##### Safety Reporting

At each study visit and online contact, participants were asked if they have experienced any AEs, change in health status, and/or had started any new medications or natural health products since the baseline visit. These data were captured using an AEs log and concomitant medication log.

### Data Analysis

#### Quantitative Analysis

##### Food Intake and Nutrient Analysis

Nutrient analysis was conducted using ESHA—The Food Processor Nutrition Analysis and Fitness Software [45] and the Canadian Nutrient File [46]. Three-day food records were manually entered by a trained research assistant and cross-checked by the coinvestigators. Averages of the 3 days of nutrient values were used in the analysis.

##### Food Frequency Analysis

To calculate usual intakes (ie, ∑ frequency weight × nutrient content), individual-level reported frequencies of consumption (ie, per day, week, month, or year) for each of the FFQ items were multiplied with standard portion sizes [35], then with nutrient calculation algorithms based on the standard portion sizes. Next, usual daily food and nutrient intakes by question were derived based on summing nutrient or food intake levels for the macro- and micronutrients, prorating their frequencies accordingly (eg, divisor of 365 for a given nutrient value if frequency of intake for the food is yearly) to provide daily nutrient or food intake values. Finally, total daily values for a given nutrient will be calculated by summing the appropriate cluster of FFQ variables that contain the nutrient of interest.

#### Descriptive Analysis

Descriptive analysis includes reporting of means (±SDs) or medians (with interquartile range) depending on continuous variable distributions. Subject characteristics between the I and C groups will be compared. The distributions of nutrient intakes will be examined and appropriately transformed if they deviate from normality.

#### Inferential Analysis

Inferential analysis includes Student *t* tests, analysis of variance (ANOVA), and Fisher exact tests to compare differences between groups and pre- and postinterventions. Analysis of the primary outcome measures involve conducting a series of repeated measures ANOVAs, comparing scores for each of the groups on the primary outcome measures (ie, food intake) at baseline and 8 weeks after receiving gene test results. The second set of analyses will involve using repeated measures ANOVAs to compare the 2 groups on measures of the different covariates. All analyses will be done on an intent-to-treat basis (last observation carried forward) using StataCorp’s STATA software [47].

#### Qualitative Analysis

Data from the focus groups will be transcribed by a professional transcriptionist and analyzed by research team members using interpretative thematic analysis. Initially, transcripts will be organized and coded as relevant passages of text. The focus group interview content will be read repeatedly to identify patterns, preliminary concepts, themes, examples, and linkages to theory [48]. Transcript codes will be compared for identifying similarities and differences through discussions among team members to refine categories and themes. Using QSR International’s NVivo [49], exemplars of coded text will be extracted. Interpretations will be reviewed by research team members and participants to check for descriptive and interpretive validity. Qualitative data will be reported based on thematic analysis derived from 3 independent reviews of the textual data.

## Results

Four hundred and seventy-eight persons expressed interest in study participation in March 2017. Participants were invited sequentially from this list. This resulted in the study coordinator contacting a total of 180 of the 478 (37.6%) interested individuals who then completed the online eligibility-screening questionnaire. Given that most interested individuals were female, attempts were made to balance the sample by sex. Seventy-three of the 180 invited individuals (40.5%) were deemed eligible. Of those who were deemed to be eligible, 55 completed the baseline health questionnaire and food records (75%). The majority of participants are female, married, and have postsecondary education. To date, 3 participants were excluded (5%). This occurred before the first visit as they did not complete all required data collection forms or were later found to be eligible. No AEs have been reported.

## Discussion

### Findings to Date

The high level of expressed interest and participant retention rate indicates that consumers are receptive to personalized nutrition approaches. The emerging science of nutrigenomics combined with personalized nutrition interventions are optimal means of providing dietary advice to the general population, genetic subgroups, and individuals. However, a demand for more sophisticated and user-friendly digital interface products that integrate a person’s phenotypic information with the person’s current nutritional status (eg, anthropometry, physical activity), current dietary intakes, and genotype-nutrition information are needed.

### Conclusions

This study proposes to compare standard and tailored personalized nutrition approaches based on gene testing and to elicit participant feedback about their experiences with the 2 types of interventions. The study results will be leveraged to generate new and tailored nutrigenomics tools that are digitally based for consumers and health professionals. The data and products derived from this investigation are intended to help advance personalized nutrition approaches that could optimize individual and population health, create efficiencies in health service delivery, and generate savings in health care expenditures.

## Appendix

Multimedia Appendix 1 Formula for calculating energy expenditure.

## Abbreviations

AE: adverse event
C: control group
DTC: direct-to-consumer
EE: energy expenditure
FFQ: Food Frequency Questionnaire
GSE: general self-efficacy
I: intervention group
HRQOL: health-related quality of life
MCS: mental component summary NHP: natural health product
PAI: physical activity index
PCS: physical component summary
RD: registered dietitian
SF8: Short-form 8
