# Supplementary material for: Comparison of Nutrigenomics Technology Interface Tools for Consumers and Health Professionals: Protocol for a Mixed-Methods Study
Source: JMIR Res Protoc. 2018 Jun 11;7(6):e115. doi: 10.2196/resprot.9846 (PMC6018240; doi:10.2196/resprot.9846)
Supplement: Multimedia Appendix 1 [file resprot_v7i6e115_app1.pdf]

Multimedia Appendix 1: Formula for calculating energy expenditure

The following is the formula for calculating energy expenditure (EE) for each activity.

The totals of all values comprise the Physical Activity Index Score (PAI).

EE = (N x D x MET value)/91, where:

N =	number of times the individual participated in the activity in the past 3 months
D =	average duration of each activity session in hours
MET =	metabolic equivalent value or the energy cost of the activity expressed in kcal/kg/hr.<br>MET reflects intensity of an activity and is expressed as multiples of the resting metabolic rate (RMR), which is approximated as 1 kcal/kg/hr: 1 MET = RMR.
91 =	number of days in 3 months that is included to convert activity in the past 3 months to an average daily value in kcal/kg/d

These totals are used to categorize individuals as inactive (PAI <1.5 kcal/kg/d),

moderately active (PAI 1.5 to <3 kcal/kg/d), and active (PAI ≥3 kcal/kg/d).

Source:

Taylor HL, Jacobs DR, Schucker B, Knudsen J, Leon AS, Debacker G. A questionnaire for leisure time physical activities. J Chronic Dis 1978;31:741-55. PMID: 748370
